# Security- and Reliability-Guaranteed Transmission Control of Time-Sensitive Physical Layer Security Systems

**DOI:** 10.3390/e25071040

**Published:** 2023-07-11

**Authors:** Jianye Li, Yunquan Dong, Chengsheng Pan

**Affiliations:** 1 School of Electronic and Information Engineering, Nanjing University of Information Science and Technology, Nanjing 210044, China; 20211249233@nuist.edu.cn (J.L.); 003150@nuist.edu.cn (C.P.); 2 School of Electronic and Information Engineering, Anhui Jianzhu University, Hefei 230009, China

**Keywords:** age of information, physical layer security, transmission control, outage probability, interception probability

## Abstract

In this paper, we consider information transmission over a three-node physical layer security system. Based on the imperfect estimations of the main channel and the eavesdropping channel, we propose reducing the outage probability and interception probability by hindering transmissions in cases where the main channel is too strong or too weak, which is referred to as an *SNR-gated transmission control scheme.* Specifically, Alice gives up its chance to transmit a packet if the estimated power gain of the main channel is smaller than a certain threshold so that possible outages can be avoided; Alice also becomes silent if the estimated power gain is larger than another threshold so that possible interceptions at Eve can be avoided. We also consider the timeliness of the network in terms of the violation probability of the peak age of information (PAoI). We present the outage probability, interception probability, and PAoI violation probability explicitly; we also investigate the trade-off among these probabilities, considering their weight sum. Our numerical and Monte Carlo results show that by using the SNR-gated transmission control, both the outage probability and the interception probability are reduced.

## 1. Introduction

In recent years, with the wide development of wireless communications, privacy and security in wireless communication networks are receiving increased attention. The main reason is that, due to the broadcasting nature of wireless transmissions, the communication between legitimate users is vulnerable to eavesdropping by malicious third parties. To enhance security, many encryption schemes have been proposed to improve the security of wireless communications. However, these encryption schemes inevitably increase the overhead of communications [1,2]. The encryption and decryption processes also require more processing resources, which impair the timeliness of the information deliveries.

In order to address security concerns, physical layer security has been proposed as a promising technology for ensuring secure wireless communications. Note that wireless channels suffer from random fading and noises. These physical characteristics can be used to encrypt the information under transmission by reducing the amount of data that can be accessed by potential eavesdropping nodes. In [3], Wyner extended Shannon’s cryptosystem in the framework of information theory in 1975. Wyner proposed the security capacity concept to evaluate the information rate that could be reliably and securely transmitted over a wireless channel. He concluded that when the eavesdropping channel quality is poorer than that of the primary channel, there exists a coding method that can provide reliable communication for the legitimate user while making it impossible for a malicious eavesdropper to decode any useful information from the captured signal. Specifically, when the instantaneous capacity of the eavesdropping channel from the source node to the eavesdropping node is smaller than the rate of the transmitted codeword, the eavesdropping node is unable to decode the source node information; thus, the legitimate transmission remains secure. However, if the instantaneous capacity of the eavesdropping channel is higher than the rate of the source codewords, the eavesdropping node would be able to successfully decode the source node’s codewords. In this case, an interception event occurs. Thus, we can reduce the *interception probability* and improve the security level by increasing the coding rate of the source. Nevertheless, *the probability of outages* (i.e., when the instantaneous capacity of the main channel is smaller than the coding rate of the source) decreases with the coding rate. In light of this contradiction, the optimal trade-off between the security and the reliability of physical layer communication has been widely investigated in [4]. Previous works, e.g., [5,6], also presented many meaningful results in the field of physical layer security. However, few works have demonstrated the ability to reduce these issues at the same time.

For communication systems, we need to consider the reliability and security of the system as well as the timeliness of communications. In previous works, the delay was widely used to measure the timeliness of communications. Recently, the emergence of AoI [7] has become a better option for precisely measuring timeliness. Specifically, the average AoI and peak AoI of systems are often used to describe the performances of real-time systems. For systems gathering information and providing status updates, however, more stringent timeliness is required. Thus, more researchers are measuring timeliness with the AoI violation probability instead of the mean AoI and peak AoI. The AoI violation probability was defined in [8] to highlight the potential damage caused by very large ages. For a single source single destination system with an FCFS serving policy, the explicit AoI violation probability has been studied in [9]. The violation probability of the peak AoI in wireless communication systems, considering practical physical layer constraints, was investigated in [10]. An approximate closed expression of the outage probability of the peak AoI exceeding a certain threshold was presented in [11]. In this paper, we investigate the timeliness of the system in terms of the violation probability of peak AoI as a measure of communication timeliness.

### 1.1. Motivations

Due to the contradiction between the outage probability and the interception probability, we are motivated to consider the following problem.

*Can we design a transmission mechanism that simultaneously reduces the outage probability and the interception probability to some extent?*

In this paper, we propose an *SNR-gated transmission control* for the source node. Specifically, the proposed method improves performance by hindering packet transmissions in slots when an outage or an interception is expected to occur. Specifically, Alice gives up its chance to transmit a packet if the estimated power gain of the main channel is smaller than a certain threshold so that possible outages can be avoided. We also consider the timeliness of transmissions in terms of the probability of peak AoI. By using a weighted sum function of the outage probability, the interception probability, and the violation probability of the peak AoI, we are able to know how these metrics vary with the transmitting power and arrival rate of the source node.

### 1.2. Related Works

Most of the existing studies on physical layer security assume that the legitimate receivers have perfect estimations of channel conditions. Since the channel estimations are often not error-free, this assumption is not very practical. In fact, channel estimation errors exist in both legitimate receivers and eavesdroppers [12,13,14]. In most channel estimation techniques, the channel state information (CSI) is obtained through a transmitted signal in the guided frequency band. However, it is usually difficult or impossible for the transmitter to know the state of the channel to the eavesdropper through estimations. In [12], it was shown that the errors in the channel estimation decrease the traversal secrecy rate. In [14], the authors investigated the optimal power allocation for artificial noise in the secure transmission, which considers the effect of the imperfect CSI on legitimate receivers.

In physical layer security-related research, errors in channel estimation reduce both the reliability and security of the system. Moreover, the correlation between the main and eavesdropping channels can also have a significant impact on system reliability and security. Most researchers assumed independent premises for the main and eavesdropping channels [15]. In actual radio environments, the proximity between the legitimate receiver and eavesdropper, along with similar surrounding scatterers, can cause a high correlation between the received signals of the two receivers. Some research has shown that this correlation can improve performance under certain conditions [16,17,18]. However, the results in [16] indicate that this correlation causes a loss in traversal secrecy capacity. Nonetheless, a strong channel correlation does not necessarily indicate a high probability of communication disruption. Reference [17] proposed that the correlation impact on the system security performance is not singular but is related to various factors, such as the average signal-to-noise ratio at the receiver, the channel gain ratio, and the set target rate.

In this paper, we propose an SNR-gated transmission control scheme for a three-node system accounting for channel estimation error and correlation between the main and eavesdropping channels. We use the outage probability as a measure of system reliability, and the interception probability as a measure of system security. With these metrics, we investigate the performances of systems with and without the SNR-gated transmission control, and with saturated or unsaturated traffic input, respectively. We also investigate the trade-off among these metrics through a weighted-sum function. By using some mathematical tools, the minimum weighted sum can be found efficiently.

### 1.3. Main Contributions

The contributions of this paper are summarized as follows.

(1) We propose a novel SNR-gated transmission control scheme to reduce the outage probability and the interception probability at the same time. By hindering transmissions with possible outages and interceptions, the power consumption of the system is also reduced, which further enhances the timeliness of the system.
(2) We consider the performances of systems with unsaturated traffic input, which is more realistic in practical engineering.
(3) We present the outage probability, the interception probability, and peak AoI violation probability explicitly, which are essential for improving and optimizing the reliability, safety, and timeliness of the system.

### 1.4. Organization

The rest of the paper is organized as follows. We present the transmission model for wireless networks in Section 2. In Section 3, we present the proposed SNR-gated transmission control scheme and derive the outage probability, the interception probability, and the violation probability of peak AoI in closed form. The Monte Carlo simulation and numerical results are presented in Section 4. Finally, we present our conclusions in Section 5.

## 2. System Model

**Figure 1:**
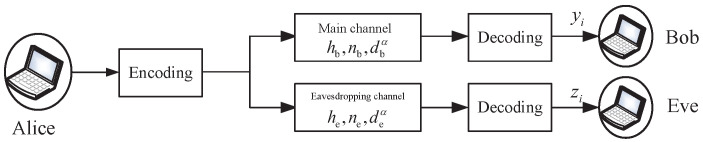
System model.

We consider a wireless network wherein Alice transmits confidential information to Bob, while a third party (Eve) attempts to eavesdrop on this confidential information, as shown in Figure 1. We denote the received signals at Bob and Eve, respectively, as $y_{i}=\sqrt{P}h_{b,i}x_{i}+n_{b,i}$, $z_{i}=\sqrt{P}h_{e,i}x_{i}+n_{e,i}$. We denote the average transmit power of Alice as *P*. We assume that the channels suffer from block Rayleigh fading and additional white Gaussian noise. Thus, the channel gain coefficients ($h_{b}$ and $h_{e}$) are zero-mean Gaussian random variables, while the noises ($n_{b}$ and $n_{e}$) are independent complex Gaussian random variables with zero mean and variances ($N_{b}^{2}$ and $N_{e}^{2}$). We denote the distance between Alice and Bob as $d_{b}$, the distance between Alice and Eve as $d_{e}$, and the path loss factor as α. The instantaneous received signal-to-noise ratio at Bob and Eve can be expressed as $\gamma_{b}=\frac{|h_{b}{|}^{2}P_{t}}{d_{b}^{\alpha }N_{b}^{2}}$, $\gamma_{e}=\frac{|h_{e}{|}^{2}P_{t}}{d_{e}^{\alpha }N_{e}^{2}}$.

We denote the signal bandwidth as *W*. Thus, the instantaneous capacities of the main channel and eavesdropping channel are given, respectively by:

(1) $$\begin{array}{c} \left\{\begin{array}{c} C_{b}=W_{b}\log_{2}\left(1+\gamma_{b}\right)\\ C_{e}=W_{e}\log_{2}\left(1+\gamma_{e}\right). \end{array}\right. \end{array}$$

### 2.1. Channel Estimation

We estimate the fading gain of the channel by using an MMSE estimator during the guided frequency transmission period [19]. The estimation of Bob’s channel gain and the estimation error are denoted by ${\hat{h}}_{b}$ and ${\tilde{h}}_{b}$, respectively. Thus, we have,

(2) $$\begin{array}{c} h_{b}={\hat{h}}_{b}+{\tilde{h}}_{b}. \end{array}$$

We assume that ${\hat{h}}_{b}$ and ${\tilde{h}}_{b}$ are independent zero-mean complex Gaussian random variables, and have [20]

(3) $$\begin{array}{c} E\left\{{\left|h_{b}\right|}^{2}\right\}=E\left\{{\left|{\tilde{h}}_{b}\right|}^{2}\right\}+E\left\{{\left|{\hat{h}}_{b}\right|}^{2}\right\}. \end{array}$$

As we know in [21], the estimation error of the channel coefficient error variance is $\beta_{b}=\sigma_{{\tilde{h}}_{b}}^{2}=\frac{1}{1+P_{p}}$, where $P_{p}$ is the pilot power. We denote the estimated SNR at Bob as ${\hat{\gamma }}_{b}=\frac{P_{t}|{\hat{h}}_{b}{|}^{2}}{d_{b}^{\alpha }N_{b}^{2}}$ and ${\tilde{\gamma }}_{b}=\frac{P_{t}|{\tilde{h}}_{b}{|}^{2}}{d_{b}^{\alpha }N_{b}^{2}}$, both of which are exponentially distributed given by random variables with the pdf:

(4) $$\begin{array}{c} \left\{\begin{array}{c} f_{{\hat{\gamma }}_{b}}\left({\hat{\gamma }}_{b}\right)=\frac{d_{b}^{\alpha }N_{b}^{2}}{P_{t}\left(1-\beta \right)}e^{-\frac{{\hat{\gamma }}_{b}d_{b}^{\alpha }N_{b}^{2}}{P_{t}\left(1-\beta \right)}},\;{\hat{\gamma }}_{b}>0\\ f_{{\tilde{\gamma }}_{b}}\left({\tilde{\gamma }}_{b}\right)=\frac{d_{b}^{\alpha }N_{b}^{2}}{P_{t}\beta }e^{-\frac{{\tilde{\gamma }}_{b}d_{b}^{\alpha }N_{b}^{2}}{P_{t}\beta }},\;{\tilde{\gamma }}_{b}>0. \end{array}\right. \end{array}$$

Due to the estimation errors, the actual instantaneous SNR at Bob can be expressed as a function of the estimated SNR and the SNR error [22]:

(5) $$\begin{array}{c} \gamma_{b}=\frac{P_{t}{\left|{\hat{h}}_{b}\right|}^{2}}{P_{t}{\left|{\tilde{h}}_{b}\right|}^{2}+1}=\frac{{\hat{\gamma }}_{b}}{{\tilde{\gamma }}_{b}+1}. \end{array}$$

### 2.2. Channel Correlation Coefficient

Since Bob and Alice are closely located, the main channel and the eavesdropping channel would be correlated. Thus, the channel gain coefficient between Alice and Eve can be expressed as in [23]:

(6) $$\begin{array}{c} h_{AE}=\xi_{AE}h_{b}+\sqrt{1-\xi_{AE}^{2}}h_{e}. \end{array}$$

In Equation (6), $h_{AE}$ is the channel gain coefficient of the main channel. $h_{b}$ and $h_{e}$ are zero-mean Gaussian random variables that are independently and identically distributed. Parameter $\xi_{AE}$ represents the correlation coefficient between the gain of the main channel and the eavesdropping channel. Moreover, the signal received by Eve can be expressed as $y_{i}=\sqrt{P}h_{AE}x_{i}+n_{E,i}$, where $n_{E,i}$ represents the additive white Gaussian noise at the eavesdropper’s receiver. Thus, the SNR at Eve can also be expressed as follows:

(7) $$\begin{array}{c} \gamma_{e}=\frac{P_{t}}{d_{e}^{2}N_{e}^{2}}\left[\xi_{AE}^{2}{\left|h_{b}\right|}^{2}+\left(1-\xi_{AE}^{2}\right){\left|h_{e}\right|}^{2}\right]. \end{array}$$

### 2.3. Traffic Model

In most previous studies, it was assumed that Alice always had packets to transmit (i.e., the saturated traffic model), so that the probability of the unsuccessful packet reception equals the probability that the channel gain is smaller than a certain threshold. In practical implementations, we need to consider the unsaturated traffic model instead. Specifically, the unsaturated model and the saturated model are explained as follows.

- Saturated traffic model: The next packet arrives immediately when the transmission of the previous packet is complete so that Alice always has packets to transmit.
- Unsaturated traffic model: The packets are generated according to a certain random process. Thus, there are slots where Alice does not have a packet to transmit. In this paper, we assume that the packets arrive with a geometric process with parameter λ. That is, Alice has a new packet with a probability λ in each slot. Thus, the average inter-arrival time would be $E\left[X_{k}\right]=\frac{1}{\lambda }$.

### 2.4. Reliability and Safety Metrics

We denote the code word transmission rate as $R_{b}$ and the confidential message rate as $R_{s}$. If the main channel capacity is less than the data rate $R_{b}$, an outage event occurs. That is, the outage probability is given by:

(8) $$\begin{array}{cc} \; & P_{\mathrm{out}}=\mathrm{Pr}\left(C_{b}<R_{b}\right). \end{array}$$

Since the transmission of a packet is successful with probability $\mu =1-P_{\mathrm{out}}$ (no outage occurs), the service time of a packet (i.e., the number of slots to transmit a packet to Bob successfully) follows a geometric distribution with parameter μ. That is,

(9) $$\begin{array}{c} \mathrm{Pr}\left\{S=j\right\}={\left(1-\mu \right)}^{j-1}\mu . \end{array}$$

Thus, we have $E\left[S\right]=\frac{1}{\mu }$.

We denote the cost of protecting message transmissions from eavesdropping as $R_{e}=R_{b}-R_{s}$. If the capacity of the eavesdropping channel satisfies $C_{e}>R_{e}$, Eve will be able to decode the message and an interception event occurs. Thus, the interception probability of the eavesdropping channel is given by:

(10) $$\begin{array}{cc} \; & P_{\mathrm{int}}=\mathrm{Pr}\left(C_{e}>R_{e}\right). \end{array}$$

Note that both the above-mentioned outage probability and interception probability are probabilities conditioned on the fact that there are always enough messages for transmission. That is:

(11) $$\begin{array}{c} \left\{\begin{array}{c} P_{\mathrm{out}}=\mathrm{Pr}\left(C_{b}<R_{b}|\mathrm{Saturation}\right)\\ P_{\mathrm{int}}=\mathrm{Pr}\left(C_{e}>R_{e}|\mathrm{Saturation}\right). \end{array}\right. \end{array}$$

### 2.5. Violation Probability of Peak AoI

The AoI of the system is defined as the length of the period between the current time and the time at which the latest received update is generated. Thus, a smaller AoI indicates fresher information. At the moment *t*, AoI is expressed as [7]:

(12) $$\begin{array}{c} \Delta (t)=t-r(t), \end{array}$$

wherein *r*(*t*)** is the timestamp of the latest update received at the receiver at time *t*. The peak AoI is defined as the age of a packet at the time it is received [24], i.e.,

(13) $$\begin{array}{c} \Delta_{p}\left(t\right)=Y_{k}+T_{k-1}, \end{array}$$

wherein $Y_{k}$ is the inter-departure time and $T_{k-1}$ is the system time of the packet.

## 3. SNR-Gated Transmission

**Figure 2:**
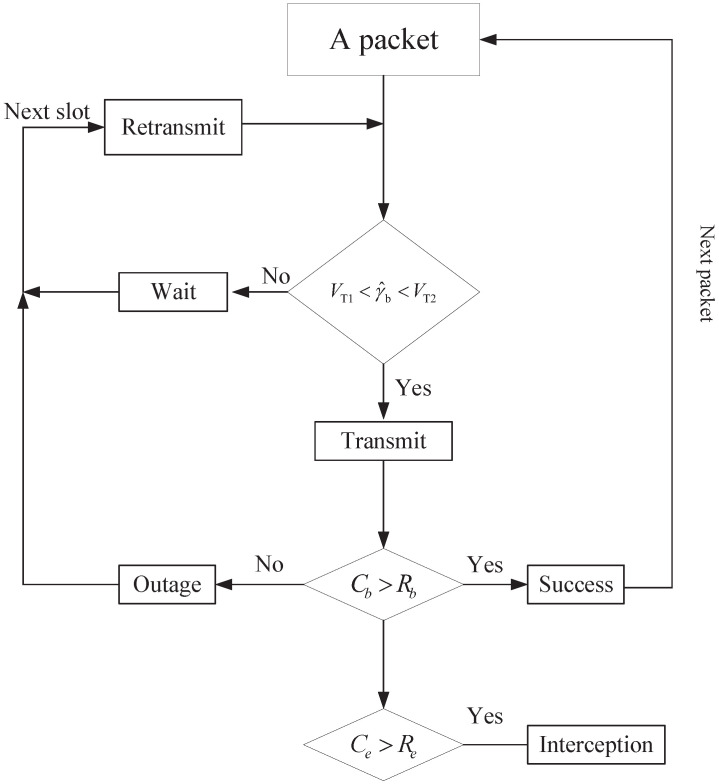
Packet transmission under SNR-based control.

In order to reduce the outage probability and the interception probability simultaneously, we propose an SNR-gated transmission control scheme in this section. Note that an outage might occur when the main channel is poor while an interception may occur if the main channel is relatively strong. Therefore, it is reasonable to control the transmission of Alice, and only perform transmission in slots where the estimated SNR of the main channel is neither too small nor too large. Figure 2 shows the transmission process of data packets under the SNR-gated transmission control.

Specifically, when the packet obtains its chance to be transmitted, we shall check if the estimated SNR of the main channel falls into the desired range $\left(V_{T1},V_{T2}\right)$. If yes, the packet will be transmitted. If not, the packet needs to wait and will be retransmitted in the next time slot. When the packet is transmitted in the current slot, an outage can still possibly occur, in which case, the packet will be retransmitted in the next time slot. A packet is considered as successfully transmitted only if no outage occurs.

### 3.1. Outage Probability

#### 3.1.1. Outage Probability under Saturated Model

We denote the lower and upper SNR thresholds of the control scheme as $V_{T1}$ and $V_{T2}$. That is, Alice performs a transmission if and only if the estimated SNR satisfies $V_{T1}<{\hat{\gamma }}_{b}<V_{T2}$. In other cases, the packet will not be transmitted; thus the probability of outage and interception is reduced. First, the outage probability of the saturated model is given by the following proposition.

**Proposition 1.**  *Regarding saturated transmission, the outage probability is expressed as follows:*

(14) $$\begin{array}{cc} \; & {\hat{P}}_{\mathrm{out}.s}=\mathrm{Pr}\left\{C_{b}<R_{b}|V_{T1}<{\hat{\gamma }}_{b}<V_{T2}\right\}\mathrm{Pr}\left\{V_{T1}<{\hat{\gamma }}_{b}<V_{T2}\right\}\\ \; & =\left(e^{-\left(\frac{V_{T1}W_{1}N_{1}d_{1}^{\alpha }}{P_{t}\left(1-\beta \right)}\right)}-e^{-\left(\frac{V_{T2}W_{1}N_{1}d_{1}^{\alpha }}{P_{t}\left(1-\beta \right)}\right)}\right)\cdot e^{-\left(\frac{W_{1}N_{1}d_{1}^{\alpha }\left(\frac{V_{T2}}{k}-1\right)}{P_{t}\beta }\right)}\\ \; & +\left(e^{-\left(\frac{W_{1}N_{1}d_{1}^{\alpha }\left(\frac{V_{T1}}{k}-1\right)}{P_{t}\beta }\right)}-e^{-\left(\frac{W_{1}N_{1}d_{1}^{\alpha }\left(\frac{V_{T2}}{k}-1\right)}{P_{t}\beta }\right)}\right)\cdot e^{-\left(\frac{V_{T1}W_{1}N_{1}d_{1}^{\alpha }}{P_{t}\left(1-\beta \right)}\right)}\\ \; & -\frac{1-\beta }{k\beta +1-\beta }e^{-\left(\frac{kW_{1}N_{1}d_{1}^{\alpha }}{P_{t}\left(1-\beta \right)}\right)}\cdot (e^{-\left(\frac{W_{1}N_{1}d_{1}^{\alpha }\left(\frac{V_{T1}}{k}-1\right)\left(k\beta +1-\beta \right)}{P_{t}\beta \left(1-\beta \right)}\right)}\\ \; & -e^{-\left(\frac{W_{1}N_{1}d_{1}^{\alpha }\left(\frac{V_{T2}}{k}-1\right)\left(k\beta +1-\beta \right)}{P_{t}\beta \left(1-\beta \right)}\right)}). \end{array}$$

**Proof.**  See Appendix A.1. □

#### 3.1.2. Outage Probability under the Unsaturated Model

Second, in the unsaturated model, Alice does not have any transmission slot with probability $\frac{\lambda }{\mu }$. The corresponding actual outage probability is given by the following proposition.

**Proposition 2.**  *Regarding unsaturated transmissions, the outage probability is expressed as follows:*

(15) $$\begin{array}{cc} \; & {\hat{P}}_{\mathrm{out}.\mathrm{us}}=\mathrm{Pr}\left\{C_{b}<R_{b}|V_{T1}<{\hat{\gamma }}_{b}<V_{T2}\right\}\mathrm{Pr}\left\{V_{T1}<{\hat{\gamma }}_{b}<V_{T2}\right\}\frac{\mu }{\lambda }\\ \; & =\left(e^{-\left(\frac{V_{T1}W_{1}N_{1}d_{1}^{\alpha }}{P_{t}\left(1-\beta \right)}\right)}-e^{-\left(\frac{V_{T2}W_{1}N_{1}d_{1}^{\alpha }}{P_{t}\left(1-\beta \right)}\right)}\right)\cdot e^{-\left(\frac{W_{1}N_{1}d_{1}^{\alpha }\left(\frac{V_{T2}}{k}-1\right)}{P_{t}\beta }\right)}\\ \; & +\left(e^{-\left(\frac{W_{1}N_{1}d_{1}^{\alpha }\left(\frac{V_{T1}}{k}-1\right)}{P_{t}\beta }\right)}-e^{-\left(\frac{W_{1}N_{1}d_{1}^{\alpha }\left(\frac{V_{T2}}{k}-1\right)}{P_{t}\beta }\right)}\right)\cdot e^{-\left(\frac{V_{T1}W_{1}N_{1}d_{1}^{\alpha }}{P_{t}\left(1-\beta \right)}\right)}\\ \; & -\frac{1-\beta }{k\beta +1-\beta }e^{-\left(\frac{kW_{1}N_{1}d_{1}^{\alpha }}{P_{t}\left(1-\beta \right)}\right)}\cdot (e^{-\left(\frac{W_{1}N_{1}d_{1}^{\alpha }\left(\frac{V_{T1}}{k}-1\right)\left(k\beta +1-\beta \right)}{P_{t}\beta \left(1-\beta \right)}\right)}\\ \; & -e^{-\left(\frac{W_{1}N_{1}d_{1}^{\alpha }\left(\frac{V_{T2}}{k}-1\right)\left(k\beta +1-\beta \right)}{P_{t}\beta \left(1-\beta \right)}\right)})\frac{\mu }{\lambda }. \end{array}$$

**Proof.**  See Appendix A.2. □

From Appendix A.2, the load factor plays a crucial role in the unsaturated transmission scenario.

### 3.2. Interception Probability

#### 3.2.1. Interception Probability under Saturated Model

Since we determine whether to transmit a packet based on the estimated SNR of the main channel, we will define and derive the interception probability of the system as follows.

**Proposition 3.**  *In saturated transmissions, the interception probability can be expressed as follows:*

(16) $$\begin{array}{cc} \; & {\hat{P}}_{\mathrm{int}.s}=\mathrm{Pr}\left\{C_{e}>R_{e}|V_{T1}<{\hat{\gamma }}_{b}<V_{T2}\right\}\mathrm{Pr}\left\{V_{T1}<{\hat{\gamma }}_{b}<V_{T2}\right\}\\ \; & =\left(e^{-\left(\frac{V_{T1}W_{1}N_{1}d_{1}^{\alpha }}{P_{t}\left(1-\beta \right)}\right)}-e^{-\left(\frac{V_{T2}W_{1}N_{1}d_{1}^{\alpha }}{P_{t}\left(1-\beta \right)}\right)}\right)\cdot e^{-\left(\frac{W_{2}N_{2}d_{2}^{\alpha }\left(\frac{V_{T2}}{k_{2}}\right)}{P_{t}\sigma_{e}}\right)}\\ \; & +\left(e^{-\left(\frac{W_{2}N_{2}d_{2}^{\alpha }V_{T1}}{P_{t}\beta k_{2}}\right)}-e^{-\left(\frac{W_{2}N_{2}d_{2}^{\alpha }V_{T2}}{P_{t}\beta k_{2}}\right)}\right)\cdot e^{-\left(\frac{V_{T1}W_{1}N_{1}d_{1}^{\alpha }}{P_{t}\left(1-\beta \right)}\right)}\\ \; & -\frac{1-\beta }{k\sigma_{e}+1-\beta }\cdot (e^{-\left(\frac{W_{2}N_{2}d_{2}^{\alpha }\left(\frac{V_{T1}}{k}\right)\left(k\sigma_{e}+1-\beta \right)}{P_{t}\sigma_{e}\left(1-\beta \right)}\right)}\\ \; & -e^{-\left(\frac{W_{1}N_{1}d_{1}^{\alpha }\left(\frac{V_{T2}}{k}\right)\left(k\sigma_{e}+1-\beta \right)}{P_{t}\sigma_{e}\left(1-\beta \right)}\right)}). \end{array}$$

**Proof.**  See Appendix A.3. □

Equation (16) indicates that the transmission of data through the channel is determined by the channel quality of the main channel. As the system is fully saturated in terms of transmission, the probability of the packet being intercepted in the channel is the probability that the capacity of the eavesdropping channel is greater than the secrecy overhead.

#### 3.2.2. Interception Probability under Unsaturated Model

**Proposition 4.**  *Regarding unsaturated transmissions, the interception probability is expressed as follows:*

(17) $$\begin{array}{cc} \; & {\hat{P}}_{\mathrm{int}.\mathrm{us}}=\mathrm{Pr}\left\{C_{e}>R_{e}|V_{T1}<{\hat{\gamma }}_{b}<V_{T2}\right\}\mathrm{Pr}\left\{V_{T1}<{\hat{\gamma }}_{b}<V_{T2}\right\}\frac{\mu }{\lambda }\\ \; & =\left(e^{-\left(\frac{V_{T1}W_{1}N_{1}d_{1}^{\alpha }}{P_{t}\left(1-\beta \right)}\right)}-e^{-\left(\frac{V_{T2}W_{1}N_{1}d_{1}^{\alpha }}{P_{t}\left(1-\beta \right)}\right)}\right)\cdot e^{-\left(\frac{W_{2}N_{2}d_{2}^{\alpha }\left(\frac{V_{T2}}{k_{2}}\right)}{P_{t}\sigma_{e}}\right)}\\ \; & +\left(e^{-(\frac{W_{2}N_{2}d_{2}^{\alpha }V_{T1}}{P_{t}\beta k_{2}})}-e^{-(\frac{W_{2}N_{2}d_{2}^{\alpha }V_{T2}}{P_{t}\beta k_{2}})}\right)\cdot e^{-\left(\frac{V_{T1}W_{1}N_{1}d_{1}^{\alpha }}{P_{t}\left(1-\beta \right)}\right)}\\ \; & -\frac{1-\beta }{k\sigma_{e}+1-\beta }\cdot (e^{-\left(\frac{W_{2}N_{2}d_{2}^{\alpha }\left(\frac{V_{T1}}{k}\right)\left(k\sigma_{e}+1-\beta \right)}{P_{t}\sigma_{e}\left(1-\beta \right)}\right)}\\ \; & -e^{-\left(\frac{W_{1}N_{1}d_{1}^{\alpha }\left(\frac{V_{T2}}{k}\right)\left(k\sigma_{e}+1-\beta \right)}{P_{t}\sigma_{e}\left(1-\beta \right)}\right)})\frac{\mu }{\lambda }. \end{array}$$

**Proof.**  See Appendix A.4. □

Therefore, after our comparative analysis and calculation, we see that, by using the SNR-gated transmission control, the reduction of the possibility of sending packets on the sender side does reduce the outage probability and interception probability. Thus, it satisfies our expectation to improve transmission reliability and security simultaneously.

### 3.3. Timeliness Analysis

We formulate the transmission over the main channel as a Geom/Geom/1 queuing policy [25] and measure the timeliness of the received packet by Bob by the violation probability of the peak AoI.

Since the traffic models of packets in the channel are different, we will analyze the timeliness in two parts. There are two commonly used packet service policies: the first-come, first-served policy (FCFS) and the last-come, first-served policy (LCFS) [24]. In this paper, we assume that the packets are served according to the FCFS rule. In the unsaturation traffic model, we assume that the state updates are generated according to a geometric process. Thus, both the inter-arrival time $X_{k}$ and service time $S_{k}$ are independent and geometrically distributed random variables, with mean $E\left[X_{k}\right]=\frac{1}{\lambda }$ and $E\left[S_{k}\right]=\frac{1}{\mu }$, respectively.

First, if the service of a packet is completed before the arrival of the next packet, the service of the next packet begins immediately upon its arrival. Second, if the service of the current packet is not completed before the next arrives, the arriving packet needs to wait before starting its service. Thus, interval $Y_{k}$ between the departures can be expressed as follows:

(18) $$\begin{array}{c} Y_{k}=\left\{\begin{array}{c} S_{k,}\;X_{k}\leq T_{k-1}\\ X_{k}-T_{k-1}+S_{k,}\;X_{k}\geq T_{k-1}. \end{array}\right. \end{array}$$

The distribution of the inter-arrival time, the service time, and the system time can be expressed as follows:

(19) $$\begin{array}{c} \left\{\begin{array}{c} \mathrm{Pr}\left(X_{k}=i\right)=\lambda {\left(1-\lambda \right)}^{i-1},i\geq 1\\ \mathrm{Pr}\left(S_{k}=i\right)=\mu {\left(1-\mu \right)}^{i-1},i\geq 1\\ \mathrm{Pr}\left(T_{k-1}=i\right)=p_{0}{\left(1-p_{0}\right)}^{i-1},i\geq 1, \end{array}\right. \end{array}$$

wherein $p_{0}=\frac{1-\mu }{1-\lambda }$.

We use the violation probability of the peak AoI as the measure of data freshness, which is expressed as follows:

(20) $$\begin{array}{c} P_{\Delta_{p}}\left(A_{T}\right)=\mathrm{Pr}\left(\Delta_{p}>A_{T}\right), \end{array}$$

where $A_{T}$ is the threshold value of the peak AoI [26].

We compare the service rate before and after using the SNR-gated transmission control. For the transmission without SNR-gated transmission control, since there is no transmission constraints, packets can be sent in each slot, with a successful probability:

(21) $$\begin{array}{c} \mu =1-P_{\mathrm{out}}. \end{array}$$

By using our SNR-gated transmission control, we only attempt to transmit a packet when the estimated SNR ${\hat{\gamma }}_{b}$ of the main channel is greater than $A_{T_{1}}$ and less than $A_{T_{2}}$. Thus, the service rate of the main channel is:

(22) $$\begin{array}{c} \hat{\mu }=\mathrm{Pr}\left\{\mathrm{send}\;\mathrm{packets}\right\}-{\hat{P}}_{\mathrm{out}.\mathrm{us}}. \end{array}$$

Since SNR-gated transmission control reduces the possibility of transmission, the probability of a successful packet delivery would also be reduced.

#### 3.3.1. Saturation Transmission Model

In this case, the server is loaded at full capacity. Specifically, a new state update arrives precisely when the last update packet leaves the queue. At this point, the inter-departure time $Y_{k}$ is equal to the service time $S_{k}$. Therefore, we have $E\left[Y_{k}T_{k}\right]=E\left[S_{k}^{2}\right]$ and $E\left[X_{k}\right]=\frac{1}{\lambda }$. Thus, we have:

(23) $$\begin{array}{c} \Delta_{p}=Y_{k}+T_{k-1}=2S_{k}. \end{array}$$

For the saturated transmission model, the service rate is mainly affected by the SNR-gated transmission control. Since the saturation transmission model generates a new packet immediately when a packet is delivered, the packet arrival rate can be considered as λ=1. Thus, we have:

(24) $$\begin{array}{c} P_{\Delta_{p}}=\mathrm{Pr}\left(\Delta_{p}>A_{T_{1}}\right)={\hat{\mu }}^{A_{T_{1}}}. \end{array}$$

#### 3.3.2. Unsaturated Transmission Model

In the unsaturated transmission model, the server is not fully loaded, so the load factor is not equal to one. In this case, $Y_{k}$ is not equal to $S_{k}$ due to the additional waiting time.

We know that our SNR-gated transmission control mainly changes the service rate to $\hat{\mu }$. By replacing μ with $\hat{\mu }$, we have

**Proposition 5.**  *The violation probability of peak AoI can be expressed explicitly as follows:*

(25) $$\begin{array}{c} P_{\Delta p}\left(A_{T}\right)=\frac{{\hat{\mu }}^{2}}{\lambda^{2}}{\left(\frac{1-\hat{\mu }}{1-\lambda }\right)}^{A_{T}-2}+\frac{{\left(1-\lambda \right)}^{A_{T}-2}{\hat{\mu }}^{2}}{{\left(\hat{\mu }-\lambda \right)}^{2}}. \end{array}$$

**Proof.**  See Appendix A.5. □

### 3.4. Joint Optimization of Safety, Reliability, and Timeliness

In this section, we jointly optimize the safety, reliability, and timeliness of the system through a weighted sum function $J=\eta_{1}P_{\mathrm{out}}+\eta_{2}P_{\mathrm{int}}+\eta_{3}P_{\Delta_{p}}$, wherein $\eta_{1},\eta_{2},\eta_{3}\in \left[0,1\right]$ are weighing coefficients and $\eta_{1}+\eta_{2}+\eta_{3}=1$. By using different weights, the performance-oriented aspects of the transmission system are different. For example, when we set $\eta_{1}$ close to unity, the reliability of the system plays the most significant role among the three dimensions. When we require a better timeliness performance, we could set $\eta_{3}$ as close to unity. We can find the optimal solution more intuitively by using the MATLAB construction function. Specifically, the optimization problem of the integrated performance can be expressed as follows:

(26) $$\begin{array}{c} \min_{P_{t},\lambda }\;\;J=\eta_{1}{\hat{P}}_{\mathrm{out}.s}+\eta_{2}{\hat{P}}_{\mathrm{int}.s}+\eta_{3}{\hat{P}}_{\Delta_{p.s}}\\ s.t,\;\;\varepsilon_{1}\leq P_{t}\leq \varepsilon_{2}\\ \;\;\;\;\;\;\;0\leq \lambda \leq 1, \end{array}$$

wherein λ is the updating rate of Alice, $\left[\varepsilon_{1},\varepsilon_{2}\right]$ is the range of power. In the saturated transmission model, we do not need to consider the optimization over λ since we have λ=1. In this case, J can be rewritten as follows:

(27) $$\begin{array}{cc} \; & \min_{P_{t}}\;\;J=\eta_{1}{\hat{P}}_{\mathrm{out}.s}+\eta_{2}{\hat{P}}_{\mathrm{int}.s}+\eta_{3}{\hat{P}}_{\Delta_{p.s}}\\ \; & s.t,\;\;\;\varepsilon_{1}\leq P_{t}\leq \varepsilon_{2}. \end{array}$$

Since the above equation is an inequality-constrained optimization problem that satisfies the Karush–Kuhn–Tucker (KKT) condition [27], we will discuss it in the following cases. By setting $\frac{\partial J}{\partial P_{t}}=0$, the optimized transmit power $P_{t}$ can be obtained. We simplify ${\hat{P}}_{\mathrm{out}.s}$, ${\hat{P}}_{\mathrm{int}.s}$, and ${\hat{P}}_{\Delta_{p.s}}$, and have the following proposition:

**Proposition 6.**  *In the saturated transmission model, the optimal transmit power is given by:*

(1) *When $\varepsilon_{1}>\frac{\ln(\frac{(\eta_{1}-\eta_{3})+1}{1-\left(\eta_{1}-\eta_{3}\right)\eta_{2}})}{\omega_{2}-\omega_{1}}$, the optimal solution is $P_{t}=\varepsilon_{1}$;*
(2) *When $\varepsilon_{2}>\frac{\ln(\frac{(\eta_{1}-\eta_{3})+1}{1-\left(\eta_{1}-\eta_{3}\right)\eta_{2}})}{\omega_{2}-\omega_{1}}$, the optimal solution is $P_{t}=\varepsilon_{2}$;*
(3) *When $\varepsilon_{1}<\frac{\ln(\frac{(\eta_{1}-\eta_{3})+1}{1-\left(\eta_{1}-\eta_{3}\right)\eta_{2}})}{\omega_{2}-\omega_{1}}<\varepsilon_{2}$, the optimal solution is $P_{t}=\frac{\ln(\frac{(\eta_{1}-\eta_{3})+1}{1-\left(\eta_{1}-\eta_{3}\right)\eta_{2}})}{\omega_{2}-\omega_{1}}$,*

*wherein $\omega_{1}$ is $\frac{V_{T1}W_{1}N_{1}d_{1}^{\alpha }}{1-\beta }$ and $\omega_{2}$ is $\frac{V_{T2}W_{1}N_{1}d_{1}^{\alpha }}{1-\beta }$.*

**Proof.**  See Appendix A.6. □

## 4. Simulation Results

In this section, we evaluate the safety, reliability, and timeliness of the system through the simulation results. We set the main channel bandwidth as $W_{1}=10^{7}$ Hz, the eavesdropping channel bandwidth as $W_{2}=10^{7}$ Hz, the distance between Alice and Bob as $d_{1}=200$ m, and the distance between Alice and Eve as $d_{2}=150$ m. We set the main channel noise power spectral density as $N_{1}=4\times 10^{-12}$ W and the eavesdropping channel noise power spectral density as $N_{2}=4\times 10^{-12}$ W. The main channel Rayleigh channel parameter $\lambda_{1}$ is set to 4 and the path loss factor α is set to 2 [28]. To verify the obtained theoretical (TH) results, we also performed corresponding Monte Carlo (MC) simulations. Specifically, we set the simulation time to 10,000 s and block spacing to $10^{-3}$. We assume that the main channel and the eavesdropping channel are correlated.

**Figure 3:**
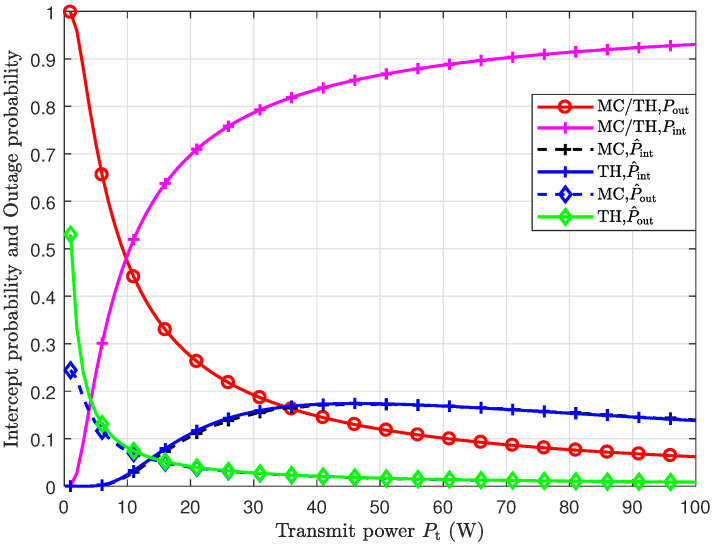
The outage probability and interception probability vary with different transmitting powers.

In Figure 3, we compare the interception probabilities and outage probabilities with and without using the SNR-gated transmission control scheme. From the figure, we can see that the outage probability decreases with the increase in transmit power $P_{t}$. More importantly, both the outage probability and interception probability are significantly decreased when the SNR-gated transmission control scheme is used. Therefore, we can conclude that the SNR-gated transmission control scheme can effectively improve reliability and safety simultaneously.

**Figure 4:**
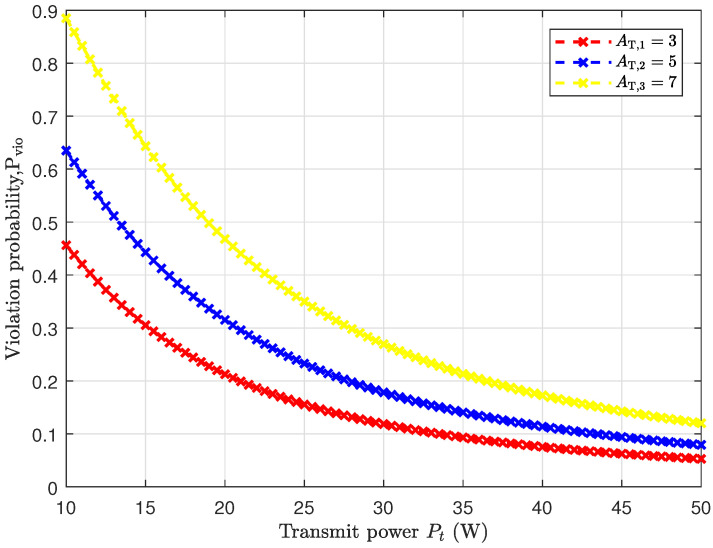
The variation of the probability of peak AoI violation with transmission power under SNR-gated transmission control. $P_{t}$ is set to 5 W. The left threshold is set to 4, the right threshold is set to 15, the pilot power is 0.7 W, and $R_{b}$ = 2.4 bps.

In Figure 4, we investigate the correlation between the transmitted power and the probability of peak AoI violation in the saturated model with SNR-gated transmission control. It is seen that as the transmission power increases, the probability of peak AoI violation decreases. By reducing the threshold of violation, the probability of peak AoI violation also decreases. This is due to the fact that increasing the threshold results in a smaller probability for the peak AoI exceeding the threshold.

**Figure 5:**
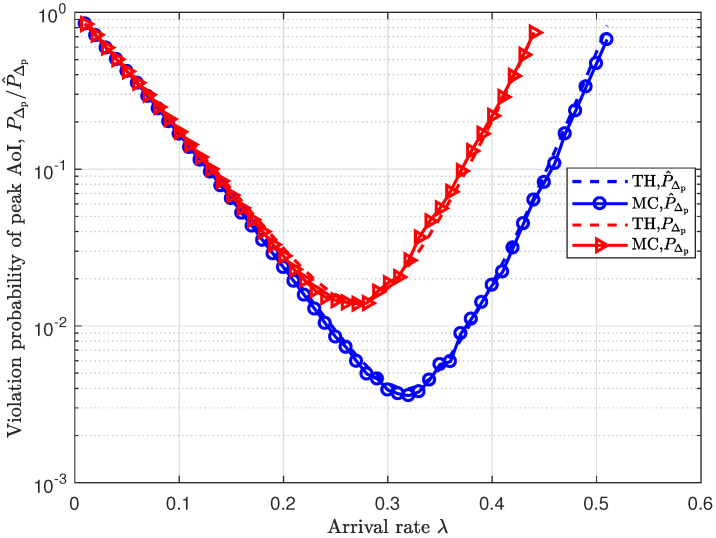
The variation in the probability of peak AoI violation with the arrival rate λ under SNR-gated transmission control and without SNR-gated transmission control.

Figure 5 shows how the violation probability of peak AoI varies with the packet arrival rate λ under the unsaturated traffic model. We observe that the violation probability decreases first and then increases. However, the peak AoI violation probability is larger than when the SNR-gated transmission control scheme is not used. This is because many transmissions are stopped, which leads to a lower service rate and, thus, degrades the timeliness.

**Figure 6:**
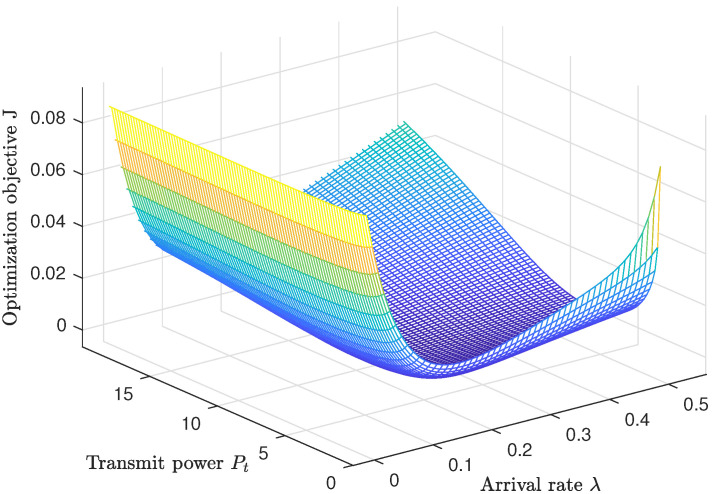
The three-dimensional graph for the weighted sum of the outage probability, interception probability, and PAoI violation probability.

In Figure 6, we present a three-dimensional graph of the weighted sum of the outage probability, interception probability, and PAoI violation probability. The *x*-axis represents the transmit power, the *y*-axis represents the arrival rate λ, and the *z*-axis represents the weighted sum of the three probabilities J. It is observed that the weighted sum has a bowl-shaped structure with a minimum point when the transmit power and the arrival rate are neither too small nor too large. This optimal point can be calculated through numerical computation.

## 5. Conclusions

In this paper, we investigate the security, reliability, and timeliness of a three-node physical layer security system, where the main channel and the eavesdropping channel are correlated. We propose an SNR-gated transmission control scheme and derive closed-form expressions for the outage probability, interception probability, and peak AoI violation probability. Our analysis and numerical results demonstrate the effectiveness of the proposed SNR-gated transmission control scheme; we explicitly derive the probabilities of data outage and interception while enhancing timeliness through the control of the data arrival rate. Moreover, we optimize the transmit power of the source node to minimize the weighted sum probability of outages, interceptions, and peak AoI violations. In the future, we plan to study the system with the adaptive rate, which is an interesting direction, as well as explore the eavesdropping channel model with relay nodes, which is another interesting research direction.

## Data Availability

Not applicable.

## Appendix A

### Appendix A.1. Proof of Proposition 1

For the actual outage probability of packets in the channel under saturated transmission, we provide the following proof:

The actual outage probability event is the product of events, where the main channel capacity is less than the encoding rate and events where data can be transmitted.

(A1) $$\begin{array}{cc} \; & {\hat{P}}_{\mathrm{out}.s}=\mathrm{Pr}\left\{C_{b}<R_{b}|V_{T1}<{\hat{\gamma }}_{b}<V_{T2}\right\}P_{suc}\\ \; & =\mathrm{Pr}\left\{W_{1}\log2\left(1+\gamma_{b}\right)<R_{b}|V_{T1}<{\hat{\gamma }}_{b}<V_{T2}\right\}P_{suc}\\ \; & =\mathrm{Pr}\left\{W_{1}\log2\left(1+\frac{{\hat{\gamma }}_{b}}{{\tilde{\gamma }}_{b}+1}\right)<R_{b}|V_{T1}<{\hat{\gamma }}_{b}<V_{T2}\right\}P_{suc}\\ \; & =\mathrm{Pr}\{V_{T1}<{\hat{\gamma }}_{b}<\min\left(k\left({\tilde{\gamma }}_{b}+1\right),V_{T2}\right)\} \end{array}$$

Since ${\hat{\gamma }}_{b}$ follows an exponential distribution, we can perform the following integration operations:

(A2) $$\begin{array}{cc} \; & =\int_{\frac{V_{T1}}{k}-1}^{\frac{V_{T2}}{k}-1}f({\tilde{\gamma }}_{b})d\left({\tilde{\gamma }}_{b}\right)\int_{V_{T1}}^{k({\tilde{\gamma }}_{b}+1)}f({\hat{\gamma }}_{b})d\left({\hat{\gamma }}_{b}\right)\\ \; & +\int_{\frac{V_{T2}}{k}-1}^{+\infty }f({\tilde{\gamma }}_{b})d\left({\tilde{\gamma }}_{b}\right)\int_{V_{T1}}^{V_{T2}}f({\hat{\gamma }}_{b})d\left({\hat{\gamma }}_{b}\right)\\ \; & =\left(e^{-\left(\frac{V_{T1}W_{1}N_{1}d_{1}^{\alpha }}{P_{t}\left(1-\beta \right)}\right)}-e^{-\left(\frac{V_{T2}W_{1}N_{1}d_{1}^{\alpha }}{P_{t}\left(1-\beta \right)}\right)}\right)\cdot e^{-\left(\frac{W_{1}N_{1}d_{1}^{\alpha }\left(\frac{V_{T2}}{k}-1\right)}{P_{t}\beta }\right)}\\ \; & +\left(e^{-\left(\frac{W_{1}N_{1}d_{1}^{\alpha }\left(\frac{V_{T1}}{k}-1\right)}{P_{t}\beta }\right)}-e^{-\left(\frac{W_{1}N_{1}d_{1}^{\alpha }\left(\frac{V_{T2}}{k}-1\right)}{P_{t}\beta }\right)}\right)\cdot e^{-\left(\frac{V_{T1}W_{1}N_{1}d_{1}^{\alpha }}{P_{t}\left(1-\beta \right)}\right)}\\ \; & -\frac{1-\beta }{k\beta +1-\beta }e^{-\left(\frac{kW_{1}N_{1}d_{1}^{\alpha }}{P_{t}\left(1-\beta \right)}\right)}\cdot (e^{-\left(\frac{W_{1}N_{1}d_{1}^{\alpha }\left(\frac{V_{T1}}{k}-1\right)\left(k\beta +1-\beta \right)}{P_{t}\beta \left(1-\beta \right)}\right)}\\ \; & -e^{-\left(\frac{W_{1}N_{1}d_{1}^{\alpha }\left(\frac{V_{T2}}{k}-1\right)\left(k\beta +1-\beta \right)}{P_{t}\beta \left(1-\beta \right)}\right)}), \end{array}$$

wherein $k=2^{\frac{R_{b}}{W_{1}}}-1$ and $\mathrm{Pr}\left\{V_{T1}<{\hat{\gamma }}_{b}<V_{T2}\right\}≜P_{suc}$.

### Appendix A.2. Proof of Proposition 2

For the actual outage probability of packets in the channel under unsaturated transmission, we provide the following proof:

(A3) $$\begin{array}{cc} \; & {\hat{P}}_{\mathrm{out}.\mathrm{us}}=\mathrm{Pr}\left\{C_{b}<R_{b}|V_{T1}<{\hat{\gamma }}_{b}<V_{T2}\right\}P_{suc}\frac{\mu }{\lambda }\\ \; & =\mathrm{Pr}\left\{W_{1}\log2\left(1+\gamma_{b}\right)<R_{b}|V_{T1}<{\hat{\gamma }}_{b}<V_{T2}\right\}P_{suc}\frac{\mu }{\lambda }\\ \; & =\mathrm{Pr}\left\{W_{1}\log2\left(1+\frac{{\hat{\gamma }}_{b}}{{\tilde{\gamma }}_{b}+1}\right)<R_{b}|V_{T1}<{\hat{\gamma }}_{b}<V_{T2}\right\}P_{suc}\frac{\mu }{\lambda }\\ \; & =\left(e^{-\left(\frac{V_{T1}W_{1}N_{1}d_{1}^{\alpha }}{P_{t}\left(1-\beta \right)}\right)}-e^{-\left(\frac{V_{T2}W_{1}N_{1}d_{1}^{\alpha }}{P_{t}\left(1-\beta \right)}\right)}\right)\cdot e^{-\left(\frac{W_{1}N_{1}d_{1}^{\alpha }\left(\frac{V_{T2}}{k}-1\right)}{P_{t}\beta }\right)}\\ \; & +\left(e^{-\left(\frac{W_{1}N_{1}d_{1}^{\alpha }\left(\frac{V_{T1}}{k}-1\right)}{P_{t}\beta }\right)}-e^{-\left(\frac{W_{1}N_{1}d_{1}^{\alpha }\left(\frac{V_{T2}}{k}-1\right)}{P_{t}\beta }\right)}\right)\cdot e^{-\left(\frac{V_{T1}W_{1}N_{1}d_{1}^{\alpha }}{P_{t}\left(1-\beta \right)}\right)}\\ \; & -\frac{1-\beta }{k\beta +1-\beta }e^{-\left(\frac{kW_{1}N_{1}d_{1}^{\alpha }}{P_{t}\left(1-\beta \right)}\right)}\cdot (e^{-\left(\frac{W_{1}N_{1}d_{1}^{\alpha }\left(\frac{V_{T1}}{k}-1\right)\left(k\beta +1-\beta \right)}{P_{t}\beta \left(1-\beta \right)}\right)}\\ \; & -e^{-\left(\frac{W_{1}N_{1}d_{1}^{\alpha }\left(\frac{V_{T2}}{k}-1\right)\left(k\beta +1-\beta \right)}{P_{t}\beta \left(1-\beta \right)}\right)})\frac{\mu }{\lambda }, \end{array}$$

where $k=2^{\frac{R_{b}}{W_{1}}}-1$.

### Appendix A.3. Proof of Proposition 3

For the actual interception probability of packets in the channel under saturated transmission, we provide the following proof:

The actual interception probability event is a product of the events where the eavesdropping channel capacity is greater than the encoding rate (which aims to prevent eavesdropping) and the events where data can be transmitted.

(A4) $$\begin{array}{cc} \; & {\hat{P}}_{\mathrm{int}.s}=\mathrm{Pr}\left\{C_{e}>R_{e}|V_{T1}<{\hat{\gamma }}_{b}<V_{T2}\right\}P_{suc}\\ \; & =\mathrm{Pr}\left\{W_{2}\log2\left(1+\gamma_{e}\right)>R_{e}|V_{T1}<{\hat{\gamma }}_{b}<V_{T2}\right\}P_{suc}\\ \; & =\mathrm{Pr}\left\{\gamma_{e}>2^{\frac{R_{e}}{W_{2}}}-1|V_{T1}<{\hat{\gamma }}_{b}<V_{T2}\right\}P_{suc}\\ \; & =\mathrm{Pr}\left\{\xi^{2}{\hat{\gamma }}_{b}+\left(1-\xi^{2}\right)\gamma_{A}>k_{1}|V_{T1}<{\hat{\gamma }}_{b}<V_{T2}\right\}P_{suc}\\ \; & =\mathrm{Pr}\left\{{\hat{\gamma }}_{b}>\frac{k_{1}-\left(1-\xi^{2}\right)\gamma_{A}}{\xi^{2}}|V_{T1}<{\hat{\gamma }}_{b}<V_{T2}\right\}P_{suc}\\ \; & =\mathrm{Pr}\{\max\left(V_{T1},\frac{k_{1}-\left(1-\xi^{2}\right)\gamma_{A}}{\xi^{2}}\right)<{\hat{\gamma }}_{b}<V_{T2}\} \end{array}$$

Since ${\hat{\gamma }}_{b}$ follows an exponential distribution, we can perform the following integration operations:

(A5) $$\begin{array}{cc} \; & =\int_{\frac{V_{T1}}{k_{2}}}^{\frac{V_{T2}}{k_{2}}}f({\tilde{\gamma }}_{b})d\left({\tilde{\gamma }}_{b}\right)\int_{V_{T1}}^{k({\tilde{\gamma }}_{b}+1)}f(\gamma_{A})d\left(\gamma_{A}\right)\\ \; & +\int_{\frac{V_{T2}}{k_{2}}}^{+\infty }f({\tilde{\gamma }}_{b})d\left({\tilde{\gamma }}_{b}\right)\int_{V_{T1}}^{V_{T2}}f(\gamma_{A})d\left(\gamma_{A}\right)\\ \; & =\left(e^{-\left(\frac{V_{T1}W_{1}N_{1}d_{1}^{\alpha }}{P_{t}\left(1-\beta \right)}\right)}-e^{-\left(\frac{V_{T2}W_{1}N_{1}d_{1}^{\alpha }}{P_{t}\left(1-\beta \right)}\right)}\right)\cdot e^{-\left(\frac{W_{2}N_{2}d_{2}^{\alpha }\left(\frac{V_{T2}}{k_{2}}\right)}{P_{t}\sigma_{e}}\right)}\\ \; & +\left(e^{-\left(\frac{W_{2}N_{2}d_{2}^{\alpha }V_{T1}}{P_{t}\beta k_{2}}\right)}-e^{-\left(\frac{W_{2}N_{2}d_{2}^{\alpha }V_{T2}}{P_{t}\beta k_{2}}\right)}\right)\cdot e^{-\left(\frac{V_{T1}W_{1}N_{1}d_{1}^{\alpha }}{P_{t}\left(1-\beta \right)}\right)}\\ \; & -\frac{1-\beta }{k\sigma_{e}+1-\beta }\cdot (e^{-\left(\frac{W_{2}N_{2}d_{2}^{\alpha }\left(\frac{V_{T1}}{k}\right)\left(k\sigma_{e}+1-\beta \right)}{P_{t}\sigma_{e}\left(1-\beta \right)}\right)}\\ \; & -e^{-\left(\frac{W_{1}N_{1}d_{1}^{\alpha }\left(\frac{V_{T2}}{k}\right)\left(k\sigma_{e}+1-\beta \right)}{P_{t}\sigma_{e}\left(1-\beta \right)}\right)}), \end{array}$$

where $k_{2}=2^{\frac{R_{e}}{W_{2}}}-1$.

### Appendix A.4. Proof of Proposition 4

For the actual interception probability of packets in the channel under unsaturated transmission, we provide the following proof:

(A6) $$\begin{array}{cc} \; & {\hat{P}}_{\mathrm{int}.\mathrm{us}}=\mathrm{Pr}\left\{C_{e}>R_{e}|V_{T1}<{\hat{\gamma }}_{b}<V_{T2}\right\}P_{suc}, \end{array}$$

owing to the fact that in an unsaturated transmission state, we need to consider the load rate of the server, so we need to multiply the load rate.

(A7) $$\begin{array}{cc} \; & =\mathrm{Pr}\left\{W_{2}\log2\left(1+\gamma_{e}\right)>R_{e}|V_{T1}<{\hat{\gamma }}_{b}<V_{T2}\right\}P_{suc}\frac{\mu }{\lambda }\\ \; & =\mathrm{Pr}\left\{\gamma_{e}>2^{\frac{R_{e}}{W_{2}}}-1|V_{T1}<{\hat{\gamma }}_{b}<V_{T2}\right\}P_{suc}\\ \; & =\mathrm{Pr}\left\{\xi^{2}{\hat{\gamma }}_{b}+\left(1-\xi^{2}\right)\gamma_{A}>k_{1}|V_{T1}<{\hat{\gamma }}_{b}<V_{T2}\right\}P_{suc}\frac{\mu }{\lambda }\\ \; & =\mathrm{Pr}\left\{{\hat{\gamma }}_{b}>\frac{k_{1}-\left(1-\xi^{2}\right)\gamma_{A}}{\xi^{2}}|V_{T1}<{\hat{\gamma }}_{b}<V_{T2}\right\}P_{suc}\frac{\mu }{\lambda }\\ \; & =\mathrm{Pr}\left\{\max\left(V_{T1},\frac{k_{1}-\left(1-\xi^{2}\right)\gamma_{A}}{\xi^{2}}\right)<{\hat{\gamma }}_{b}<V_{T2}\right\}\frac{\mu }{\lambda }\\ \; & =\left(e^{-\left(\frac{V_{T1}W_{1}N_{1}d_{1}^{\alpha }}{P_{t}\left(1-\beta \right)}\right)}-e^{-\left(\frac{V_{T2}W_{1}N_{1}d_{1}^{\alpha }}{P_{t}\left(1-\beta \right)}\right)}\right)\cdot e^{-\left(\frac{W_{2}N_{2}d_{2}^{\alpha }\left(\frac{V_{T2}}{k_{2}}\right)}{P_{t}\sigma_{e}}\right)}\\ \; & +\left(e^{-(\frac{W_{2}N_{2}d_{2}^{\alpha }V_{T1}}{P_{t}\beta k_{2}})}-e^{-(\frac{W_{2}N_{2}d_{2}^{\alpha }V_{T2}}{P_{t}\beta k_{2}})}\right)\cdot e^{-\left(\frac{V_{T1}W_{1}N_{1}d_{1}^{\alpha }}{P_{t}\left(1-\beta \right)}\right)}\\ \; & -\frac{1-\beta }{k\sigma_{e}+1-\beta }\cdot (e^{-\left(\frac{W_{2}N_{2}d_{2}^{\alpha }\left(\frac{V_{T1}}{k}\right)\left(k\sigma_{e}+1-\beta \right)}{P_{t}\sigma_{e}\left(1-\beta \right)}\right)}\\ \; & -e^{-\left(\frac{W_{1}N_{1}d_{1}^{\alpha }\left(\frac{V_{T2}}{k}\right)\left(k\sigma_{e}+1-\beta \right)}{P_{t}\sigma_{e}\left(1-\beta \right)}\right)})\frac{\mu }{\lambda }, \end{array}$$

where $k_{2}=2^{\frac{R_{e}}{W_{2}}}-1$.

### Appendix A.5. Proof of Proposition 5

For $X_{k}\leq T_{k-1}$, we have $Y_{k}=S_{k}$. Thus, we can obtain the following:

(A8) $$\begin{array}{cc} \; & P_{\Delta_{p}}\left(A_{T}\right)=\mathrm{Pr}\left(\Delta_{peak}A_{T}\right)\\ \; & =\mathrm{Pr}\{T_{k-1}+S_{k}>A_{T}|X_{k}\leq T_{k-1}\}\\ \; & =\frac{\mathrm{Pr}\{T_{k-1}>{\left\{X_{k},A_{T}-S_{k}\right\}}_{\max}\}}{\mathrm{Pr}\{X_{k}\leq T_{k-1}\}}, \end{array}$$

When $X_{k}>A_{T}-S_{k}$, the above equation is meaningless; thus, we can obtain the following:

(A9) $$\begin{array}{cc} \; & \frac{\mathrm{Pr}\{T_{k-1}>{\left\{X_{k},A_{T}-S_{k}\right\}}_{\max}\}}{\mathrm{Pr}\{X_{k}\leq T_{k-1}\}}\\ \; & =\frac{\mathrm{Pr}\{T_{k-1}>A_{T}-S_{k}\}}{\mathrm{Pr}\{X_{k}\leq T_{k-1}\}}\\ \; & =\frac{\sum_{i=1}^{\infty }\mathrm{Pr}\left\{S_{k}=i\right\}\mathrm{Pr}\left\{T_{k-1}+i>A_{T}\right\}}{\mathrm{Pr}\{X_{k}\leq T_{k-1}\}}\\ \; & =\frac{\sum_{i=1}^{\infty }\mathrm{Pr}\left\{S_{k}=i\right\}(1-\mathrm{Pr}\left\{T_{k-1}+i\leq A_{T}\right\})}{\mathrm{Pr}\{X_{k}\leq T_{k-1}\}}\\ \; & ={\sum_{i=1}^{\infty }\mathrm{Pr}\left\{S_{k}=i\right\}\left(\frac{1-\mu }{1-\lambda }\right)}^{A_{T}-i-1}={\left(\frac{\mu }{\lambda }\right)}^{2}{\left(\frac{1-\mu }{1-\lambda }\right)}^{A_{T}-2}. \end{array}$$

For $X_{k}\geq T_{k-1}$, we have $Y_{k}=S_{k}+X_{k}-T_{k-1}$. Thus, can obtain the following:

(A10) $$\begin{array}{cc} \; & \frac{\mathrm{Pr}\{T_{k-1}+Y_{k}>A_{T}\}}{\mathrm{Pr}\{X_{k}>T_{k-1}\}}\\ \; & =\frac{\mathrm{Pr}\{X_{k}>A_{T}-S_{k}\}}{\mathrm{Pr}\{X_{k}>T_{k-1}\}}\\ \; & =\frac{\sum_{i=1}^{\infty }\mathrm{Pr}\left\{S_{k}=i\right\}\mathrm{Pr}\left\{X_{k}+i>A_{T}\right\}}{\mathrm{Pr}\{X_{k}>T_{k-1}\}}\\ \; & =\sum_{i=1}^{\infty }\mu {\left(1-\mu \right)}^{i-1}{\left(1-\lambda \right)}^{A_{T}-i-1}=\frac{{\left(1-\lambda \right)}^{A_{T}-2}\mu^{2}}{{\left(\mu -p\right)}^{2}}. \end{array}$$

### Appendix A.6. Proof of Proposition 6

For the optimization issues, we provide the following proofs:

(A11) $$\begin{array}{cc} \; & \min_{P_{t}}\;\;J=\eta_{1}{\hat{P}}_{\mathrm{out}.s}+\eta_{2}{\hat{P}}_{\mathrm{int}.s}+\eta_{3}{\hat{P}}_{\Delta_{p.s}}\\ \; & s.t,\;\;\varepsilon_{1}\leq P_{t}\leq \varepsilon_{2}. \end{array}$$

In order to simplify the calculation, we can simplify:

(A12) $$\begin{array}{cc} \; & {\hat{P}}_{\mathrm{out}.s}\approx \left(e^{-(\frac{V_{T1}W_{1}N_{1}d_{1}^{\alpha }}{P_{t}\left(1-\beta \right)})}-e^{-(\frac{V_{T2}W_{1}N_{1}d_{1}^{\alpha }}{P_{t}\left(1-\beta \right)})}\right)\cdot e^{-(\frac{W_{1}N_{1}d_{1}^{\alpha }\left(\frac{V_{T2}}{k}-1\right)}{P_{t}\beta })},\\ \; & {\hat{P}}_{\mathrm{int}.s}\approx \left(e^{-(\frac{V_{T1}W_{1}N_{1}d_{1}^{\alpha }}{P_{t}\left(1-\beta \right)})}-e^{-(\frac{V_{T2}W_{1}N_{1}d_{1}^{\alpha }}{P_{t}\left(1-\beta \right)})}\right)\cdot e^{-(\frac{W_{2}N_{2}d_{2}^{\alpha }\left(\frac{V_{T2}}{k_{2}}\right)}{P_{t}\sigma_{e}})},\\ \; & {\hat{P}}_{\Delta_{P}}\approx \left(1-{\hat{P}}_{\mathrm{out}.s}\right). \end{array}$$

We denote $\omega_{1}=\frac{V_{T1}W_{1}N_{1}d_{1}^{\alpha }}{\left(1-\beta \right)}$, $\omega_{2}=\frac{V_{T2}W_{2}N_{2}d_{2}^{\alpha }}{\left(1-\beta \right)}$ and $\omega_{3}=\frac{V_{T2}W_{2}N_{2}d_{2}^{\alpha }}{\sigma_{e}}$. From $\frac{\partial J}{\partial P_{t}}=0$, we know that:

(A13) $$\begin{array}{cc} \; & \left(e^{-\frac{\omega_{1}}{P_{t}}}\frac{\omega_{1}}{{P_{t}}^{2}}-e^{-\frac{\omega_{2}}{P_{t}}}\frac{\omega_{2}}{{P_{t}}^{2}}\right)\left(\left(\eta_{1}-\eta_{3}\right)e^{-\frac{\omega_{2}}{P_{t}}}+\eta_{2}e^{-\frac{\omega_{3}}{P_{t}}}\right)\\ \; & +\left(e^{-\frac{\omega_{1}}{P_{t}}}-e^{-\frac{\omega_{2}}{P_{t}}}\right)\left(e^{-\frac{\omega_{2}}{P_{t}}}\frac{\omega_{2}}{{P_{t}}^{2}}-e^{-\frac{\omega_{3}}{P_{t}}}\frac{\omega_{3}}{{P_{t}}^{2}}\right))=0, \end{array}$$

where $\omega_{{}_{2}}\approx \omega_{3}$; thus, we can conclude the following:

(A14) $$\begin{array}{c} P_{t}=\frac{\ln(\frac{(\eta_{1}-\eta_{3})+1}{1-\left(\eta_{1}-\eta_{3}\right)\eta_{2}})}{\omega_{2}-\omega_{1}}. \end{array}$$
